# RPIOSL: construction of the radiation transfer model for rice leaves

**DOI:** 10.1186/s13007-023-01127-6

**Published:** 2024-01-03

**Authors:** Shuang Xiang, Zhongyu Jin, Jinpeng Li, Fenghua Yu, Tongyu Xu

**Affiliations:** 1 College of Information and Electrical Engineering, Shenyang Agricultural University, Shenyang, China; 2 Key Laboratory of Intelligent Agriculture in Liaoning Province, Shenyang, China

**Keywords:** Rice, Radiative transfer, PIOSL, Hyperspectral, Reflectance

## Abstract

The radiative transfer model of vegetation leaves simulates the transmission mechanism of light inside the vegetation and simulates the reflectivity of blades according to the change law of different components in the process of plant growth. Based on the PIOSL model, this paper combines PIOSL with the structure of rice leaves to construct a radiation transfer model for rice leaves. The parameters of each layer of the RPIOSL model are determined by the Non-dominated Sorting Genetic Algorithm-III. (NSGA-III.) algorithm. The reflectance spectra of 218 rice leaf samples in different periods were simulated using the RPIOSL model. The results show that the mean (RMSE) between the simulated and measured spectra of the constructed RPIOSL model is 0.1074, which is 0.0191 lower than that of the PROSPECT model. Among them, the spectral simulation effect of RPIOSL model in yellow and red light band is the best, and the RMSE at tillering period, jointing period, heading period and grouting period are 0.0584, 0.0576, 0.0724 and 0.0820, respectively. Therefore, the establishment of the RPIOSL model can accurately describe the interaction mechanism between light, which is of great significance for the rapid acquisition of rice growth information and accurate crop management.

## Introduction

Hyperspectral remote sensing technology is a significant approach to obtain large-scale crop and its change information, and has the advantages of nondestructive, noninvasive, fast and cost-effective. It has been widely used to monitor crop growth [16], map vegetation area [27], and estimate crop yield [20]. In recent years, problems such as environmental pollution have led to a reduction in grain production. Rice is an important source of grain in China. Accurate monitoring of rice growth is an essential means to ensure food security and agricultural production. A rice nitrogen inversion model is established by retrieving hyperspectral reflectance data from UAV by Du. [4]. The rice canopy was treated under different shading conditions, and it was found that diffuse reflectance would lead to the enhancement of rice canopy reflectance spectrum by Zhang [35]. The residual block and convolution block were combined to identify rice hyperspectral information in order to improve the effect of rice quality detection by Men [19]. The hyperspectral inversion approach has been widely used for quantitative detection of rice and other vegetation’s physical and chemical parameters [31]. However, hyperspectral data contains a large amount of spectral information, and there is a high degree of multicollinearity in the high-dimensional band. Therefore, proper spectral processing methods should be selected in the quantitative detection of rice physical and chemical parameters. At present, there are mainly two spectral feature analysis methods for physicochemical component estimation, which are the empirical method and the physical model inversion method. Empirical methods such as wavelet analysis [18] and building vegetation index [36] are relatively easy to achieve Inversion Modeling by collecting certain sample data from field experiments, but they lack a certain theoretical support. The model lacks the mechanism of interaction between light and the internal configuration of leaves, which leading to low generalization of the model.

By simulating the transport mechanism of light in vegetation, the relationship between the biochemical parameters of vegetation leaves and leaf reflectivity and transmittance is established according to the change law of different components and leaf spectra during plant growth. The traditional empirical method detects the spectral and biochemical parameters of leaves through a large number of manpower and material resources, and the applicability of the usually constructed model is not strong, while the radiation transmission model does not change with the study area, and it has stronger applicability to vegetation remote sensing physics [12]. Radiation transport models have been derived into a variety of types, mainly divided into PLATE model, N-Flux model, Compace specical model, radiation transport equation, stochastic model, etc. The PLATE model is applicable to the radiative transfer modeling of most rice leaves and other vegetation leaves and has been widely used since it was proposed in 1969 [1]. The most typical model is the PROSPECT model. The PROSPECT model, which was proposed by Jacquemoud [13] on the basis of a plate, can accurately calculate the spectrum of vegetation leaves. The PROSPECT model contains four parameters, namely structural parameters, chlorophyll, water, and dry matter. Later, carotenoids are separated from chlorophyll and constructed the PROSPECT-5 model was constructed by Féret [8]. Anthocyanins are added to the PROSPECT-5 model and the proposed PROSPECT-D model, which realized the simulation of optical characteristics of leaves from vegetation by Féret [9]. Later, the PROSPECT-PRO model is proposed by Féret [10], which can decompose dry matter into proteins and carbonaceous compounds (CBC). PROSPECT and its improved versions have been widely used in spectral simulation of vegetation leaves and inversion of physical and chemical parameters. The stability of PROSPECT model was evaluated by Zhai [37] in the process of retrieving of leaf chlorophyll content. For all datasets in the study, the performance of the PROSPECT model was superior to that of the random forest (RF) model. Copper content was added to the input parameters on the PROSPECT-5 model by Zhang [38] to simulate the spectra of leaves under copper stress, and the simulation error of the model at key wavelengths was close to zero. Although the PROSPECT model has been improved many times and has been verified by a large number of scholars in the aspects of reflectivity simulation and biochemical content monitoring of various plant leaves, the PROSPECT model assumes that the distribution of internal tissues and absorbed substances in leaves is uniform, while the real vegetation leaves do not have a uniform distribution structure, and the materials in the real vegetation leaves, such as chlorophyll and carotenoids, will gather in the upper or lower layers of leaves with the growth of vegetation, so it is unreasonable to simply assume that the leaves are evenly distributed inside [24]. For this kind of leaves, if we study according to the hypothesis of the PROSPECT model, there will be some errors.

Some scholars have also studied differences in optical properties caused by the layered structure of the blade. The FASPECT model proposed by Jiang [14] views the blade as a four-layer structure. The upper and lower surfaces of the blade are symmetrically treated to describe the different optical properties of the upper and lower surfaces. Additionally, a simpler radiative transfer model ISPECT proposed by Shi [21] based on the FASPECT model, which accounted for the difference in optical properties observed from the upper and lower parts of the blade. The model had fewer parameters and accurately estimated the LMA. The internal structure of leaves was stratified by Yu [33] adopted the same idea as FASPECT model to construct PIOSL model. Bald eagle optimization algorithm (BE) was used to determine the structural parameters of two layers of leaves, the proportion of chlorophyll, water and dry matter in PIOSL model. It can be seen that the construction of radiation transport model in line with the real structure of leaves is of great significance for the simulation of optical characteristics and biochemical parameter inversion of vegetation leaves. Since the internal tissue distribution of vegetation leaves changes with photosynthesis or living environment, there are also certain differences in the distribution of internal substances of leaves for different vegetation types.

Rice, an significant food crop, is taken into consideration in this paper. The PIOSL model is modified by adjusting its parameters, resulting in the establishment of RPIOSL, a radiation transfer model for rice leaves. The construction of RPIOSL model is helpful to master the reflection, scattering and transmission process of light in rice leaves, and it is of great significance to establish a more accurate hyperspectral detection method for rice physical and chemical parameters.

## Materials and methods

### Experimental design

**Fig. 1:**
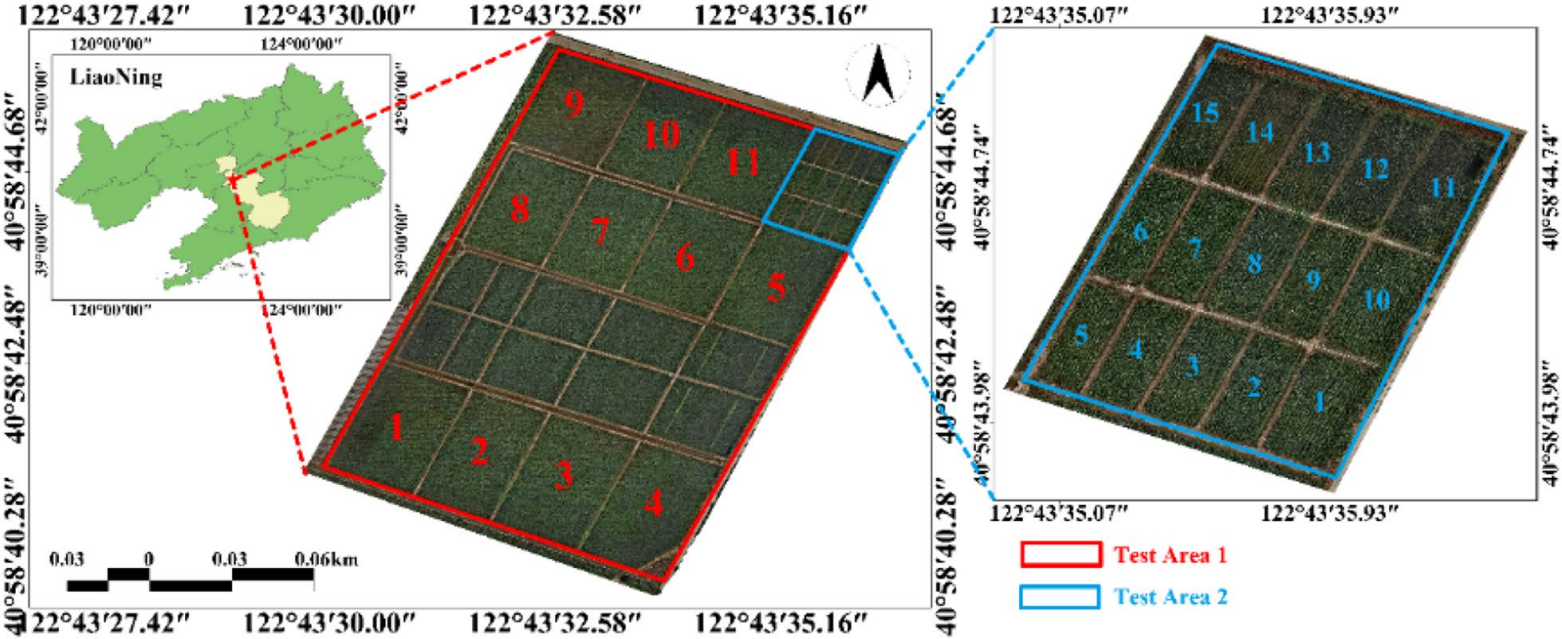
Distribution of the experimental area

The experiment was implemented at the Shenyang Agricultural University precision agricultural aviation research base (40° 58 ′45.39'n, 122° 43′ 47.0064'E), Gengzhuang Town, Haicheng City, Anshan City, Liaoning Province from June to September 2023. Due to the limitation of the conditions in the test area, the rice variety planted in the experimental area is Shennong 9816. It is divided into two experimental areas, experimental area 1 is divided into 11 areas, each area of 660 ${{\text{m}}}^{2}$, and experimental area 2 is divided into 15 areas, each area of 5 × 8 = 40 ${{\text{m}}}^{2}$, as shown in Fig. 1. The field management of the two communities is consistent. Field sampling was carried out from tillering period to heading period, with a sampling interval of 4 days. 15 plots were randomly selected in experimental areas 1 and 2 for each sampling, and a representative hole rice was selected to obtain leaf reflectance spectra and biochemical parameters. 218 groups of samples were collected in the experiment.

### Acquisition of the experimental area

**Fig. 2:**
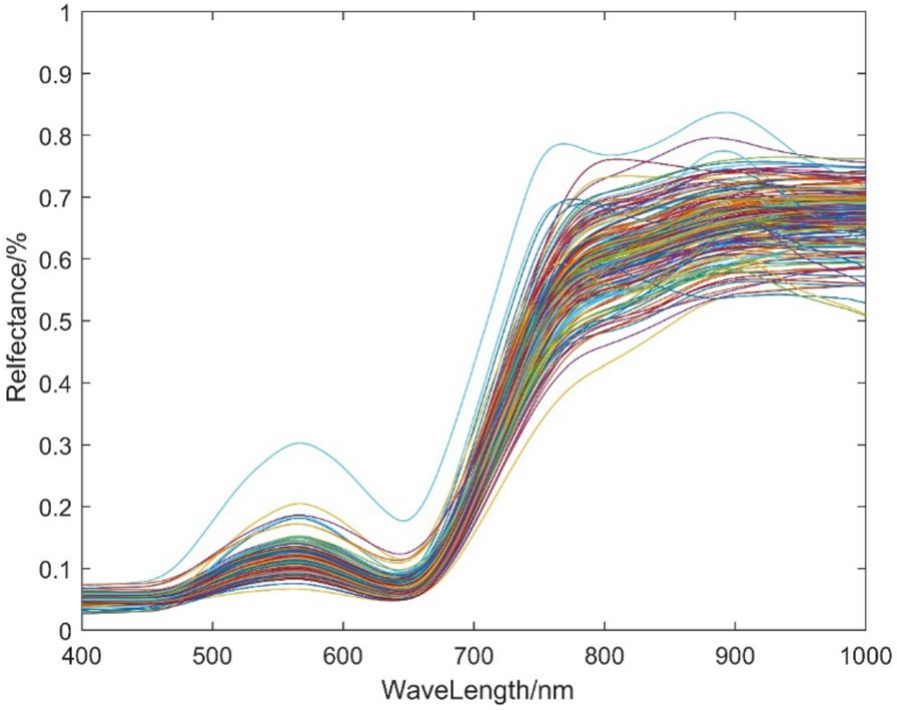
Reflectance spectrum curve after SG smoothing

The model of the AOPUTIANCHENG ground object spectrometer is the ATP9110 ground object spectrometer V2.2. The reflectance spectrum band range is 400–1000 nm, the resolution is 1 nm, and the number of effective bands is 601. In the process of calculating the model error in this paper, only the band of 400–1000 nm is calculated. Before measurement, the spectrometer was corrected with a white board and then a plant was randomly selected from each hole to measure the reflectance spectrum of the rice leaf. The whiteboard must be corrected every 10 min. Finally, the reflectance spectrum is resampled with the MATLAB r2023a software, and the spectral resolution is reduced to 1 nm. After that, the resampled spectrum is SG smoothed, and the smoothed spectrum curve is shown in Fig. 2.

### Measurement of biochemical parameters

The biochemical parameters measured in this experiment include chlorophyll, equivalent water thickness, and dry matter content. The chlorophyll content is determined by spectrophotometry. The absorbance value of chlorophyll extract at the maximum absorption wavelength is determined by a spectrophotometer, and then the chlorophyll content in the extract is calculated by the Lambert–Beer law. The experimental steps are as follows:

1. Take a rice leaf, measure the maximum length and width of the leaf, use formula (1) to calculate the leaf area of each leaf, and get the average leaf area of the sample.
  1 $${\text{A}}\, = \,{\text{a}}\, * \,{\text{b}}\, * \,0.7746$$

Among them, a is the maximum length of leaves, B is the maximum width of leaves, and 0.7746 is the leaf area correction coefficient of rice leaves [22].

2. Weigh around 0.1 g of fresh cut sample, place it in the grinding container, add a bit of quartz sand, grind it into a uniform paste, continue to grind until the tissue is obviously white, and put it on the table to stand still.
3. Put a piece of filter paper in the funnel, wet the filter paper with a small amount of ethanol solution, slowly inject the extract into the funnel with a glass rod, filter it into a 50 ml test tube, rinse the filtered research body, research rod and glass rod with a small amount of ethanol solution, and finally pour them into the funnel together.
4. Add a proper amount of ethanol solution into the 50 ml test tube by pouring in a beaker. When reaching the 50 ml scale line, use a rubber dropper to fix the volume to 50 ml and shake it evenly.
5. A small amount of chlorophyll extract was sucked by a rubber dropper and dripped into a colorimetric cup with a light diameter of 1 cm. After dark treatment and standing for 24 h, take 95% ethanol blank, use the uv1800pc ultraviolet visible spectrophotometer to quantify the absorbance of the liquid fraction containing chlorophyll at a wavelength of 665 nm and 649 nm, calculate the concentration leves of chlorophyll a and chlorophyll b using formulas (2) and (3), and then convert it $\mathrm{\mu g}\cdot {{\text{cm}}}^{-2}$ is the unit.
  2 $$C_{a} \, = \,13.95\, * \,{\text{A}}665\, - \,6.88\, * \,{\text{A}}649$$
  3 $$C_{b} \, = \,24.96\, * \,{\text{A}}649\, - \,7.32\, * \,{\text{A}}665$$
6. Weigh the fresh weight of the remaining blades, ${M}_{fresh}$, and then dry them at 70° C to constant weight after 30 min at 105° C. reweigh the dried disc to obtain ${M}_{dry}$ and calculate the equivalent water thickness of the blades using formula (4).
  4 $${\text{Cw}}\, = \,\left( {M_{dry} \, - \,M_{fresh} } \right)/A_{leaf}$$
7. Use formula (5) to calculate the dry matter content of leaves.
  5 $$C_{m} \, = \,M_{dry} /A_{leaf}$$

Table 1 gives the measurement results of biochemical parameters of rice samples collected in different periods.

**Table 1:** Statistical results of the biochemical parameters of rice leaves

	Number of samples	Chlorophyll ($\mathrm{\mu g}\cdot {{\text{cm}}}^{-2}$)			Equivalent water thickness (${\text{g}}\cdot {{\text{cm}}}^{-2}$)			Dry matter ($\mathrm{\mu g}\cdot {{\text{cm}}}^{-2}$)		
		Max	Min	Mean	Max	Min	Mean	Max	Min	Mean
Tillering period	38	110.6944	6.1493	29.3668	0.0596	0.0024	0.0231	0.0131	0.0013	0.0068
Jointing period	60	57.9034	6.2138	28.4896	0.1498	0.0038	0.0107	0.0130	0.0022	0.0040
Heading period	60	61.8711	8.6600	26.5721	0.0203	0.0042	0.0103	0.0129	0.0026	0.0055
Grouting period	60	57.6898	7.5908	31.1441	0.0169	0.0036	0.0078	0.0073	0.0023	0.0045

### RPIOSL model optimization algorithm

In this paper, NSGA-III is used to find the optimal parameters in RPIOSL model. Four parameters are optimized, including the structural parameters N1 and N2 of each layers, the proportion of chlorophyll content and dry matter ${Cab}_{12}$、${Cm}_{12}$. The principle of optimizing RPIOSL model by NSGA-III algorithm is to add new reference points to NSGA-II to maintain the diversity of population, and retain the nearest and non-dominant population individuals.

The following are the t-generation steps of NSGA- III:

${{\text{P}}}_{t}$ is the parent of generation t and its size is N. Its offspring is${Q}_{t}$, and its size is N.

Step 1: combine the offspring and parents together: ${R}_{t}={R}_{t}\cup {R}_{t}$ with a size of 2N, and select N individuals from them. In order to achieve this selection process, first divide ${R}_{t}$ into multiple non- dominated layers (${F}_{1}, {F}_{2},\cdot \cdot \cdot$) through non-dominated sorting, and then construct a new population ${S}_{t}$ from ${F}_{1}$ until its size reaches n or exceeds n for the first time. The last layer is designated as the first layer. Solutions above level l + 1 will be eliminated. In most cases, the last accepted layer (layer III) is only partially accepted.

Step 2: Populations are divided into multiple nondominating layers in order of nondominance. Construct ${S}_{t}$ starting from ${F}_{1}$. If $\left|{S}_{t}\right|={\text{N}}$, the following operation is not required, that is, ${P}_{t+1}={S}_{t}$. If $\left|{S}_{t}\right|>{\text{N}}$, part of the next generation is generated from ${P}_{t+1}={U}_{i=1}^{l-1}{F}_{i}$, and the rest ${\text{K}}={\text{N}}-|{P}_{t+1}|$ is selected from ${F}_{l}$.

Step 3: Determine the position of the reference point on the hyperplane. In NSGA-III, the reference point is predefined to increase the comprehensiveness of the solution. In this paper, the reference points are predefined by the boundary crossing construction method which sets the reference points on the same hyperplane [5].

Step 4: adaptive normalization of the individual population. First, calculate the minimum value of each objective function ${U}_{\tau =0}^{t}{S}_{\tau }$ in each dimension, ${{\text{z}}}_{i}^{min},i=\mathrm{1,2},\mathrm{3,4},\dots M$ to establish the ideal point of the current population in the target space $\overline{z }=({z}_{1}^{min},{z}_{2}^{min},\dots {z}_{M}^{min})$. Subtract the coordinate origin, the objective function value ${f}_{i}$ of each individual from ${z}_{i}^{min}$ transform the ideal point to the origin of coordinates and obtain the converted objective function value ${f}_{i}^{\mathrm{^{\prime}}}={f}_{i}\left(x\right)-{z}_{i}^{min}$. Determine the pole of each coordinate dimension after the transformation and record the intersection of the i-th dimension on the target axis as ${a}_{i}$, then the i-dimensional objective function can be normalized to Eq. (6):

6 $$f_{i}^{n} \, = \,{\raise0.7ex\hbox{${f_{i}{\prime} \left( x \right)}$} \!\mathord{\left/ {\vphantom {{f_{i}{\prime} \left( x \right)} {a_{i} }}}\right.\kern-0pt} \!\lower0.7ex\hbox{${a_{i} }$}}{\text{i}} = 1,2,3,4 \ldots {\text{M}}$$

At this time, the intercept of the normalized target axis is ${f}_{i}^{n}=1$, and the hyperplane established by using these intercept points meets ${\sum }_{i=1}^{M}{f}_{i}^{n}=1$. The set of structured reference points established by the system method will be uniformly distributed in the normalized hyperplane. In each generation of the algorithm process, the calculated poles will be used to complete the normalization step and establish the hyperplane. Therefore, NSGA-III can adaptively maintain the diversity of the population.

When optimizing the RPIOSL model, the fitness function is modified to Eq. (7):

7 $${\text{y}}\, = \,\mathop \sum \limits_{l = 400}^{1000} \left| {R_{mod,l} \, - \,R_{{meas,^{\prime}}} } \right|/R_{meas,l}$$

where, l is the number of bands, ranging from 400 to 1000 nm, ${R}_{mod,l}$ and ${R}_{meas,\mathrm{^{\prime}}}$ are the simulated and measured reflectivity apectra at band l, respectively.

### Accuracy evaluation of the model

In order to evaluate the accuracy of the RPIOSL model, this paper uses the (RMSE) to calculate the difference between the simulated reflectance and the measured reflectance between 400 nm and 1000 nm for each sample:

8 $${\text{RMSE}}\, = \,\frac{1}{{R_{meas} }}\sqrt {\mathop \sum \limits_{j = 1}^{n} \left( {R_{meas,j} \, - \,R_{mod,j} } \right)^{2} }$$

where, ${R}_{meas,j}$ and ${R}_{mod,j}$ are the measured reflectivity and simulated reflectivity of a blade j, and n is the number of samples.

## Construction principle of the RPIOSL model

**Fig. 3:**
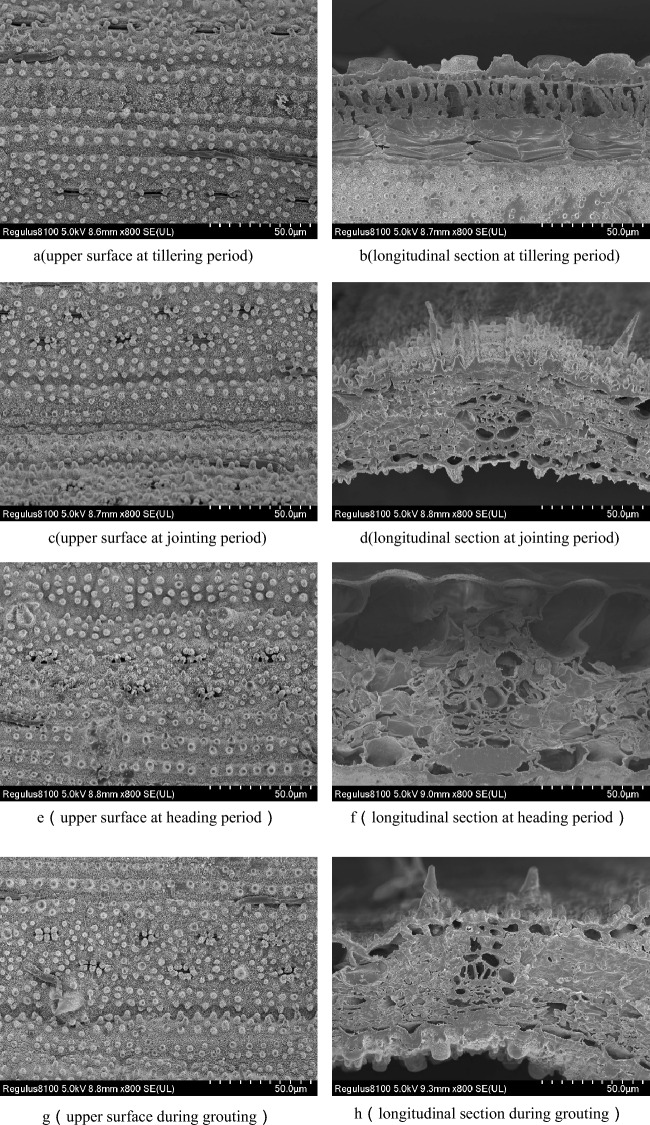
Scanning electron microscope of rice leaves

Each version of the PROSPECT model considers that the leaves are evenly distributed plates inside, while the different tissues and absorbent substances inside the real vegetation leaves have a certain stratification phenomenon, which changes with the different growth environments or growth cycles of the vegetation. We scanned the electron microscopic images of rice leaves at different stages of growth and development, including tillering period, jointing period, heading period and grouting period, and found that there was obvious stratification on the longitudinal cutting surface of rice leaves, as shown in Fig. 3. The stratification phenomenon of rice leaves at tillering period and heading period is more obvious.

**Fig. 4:**
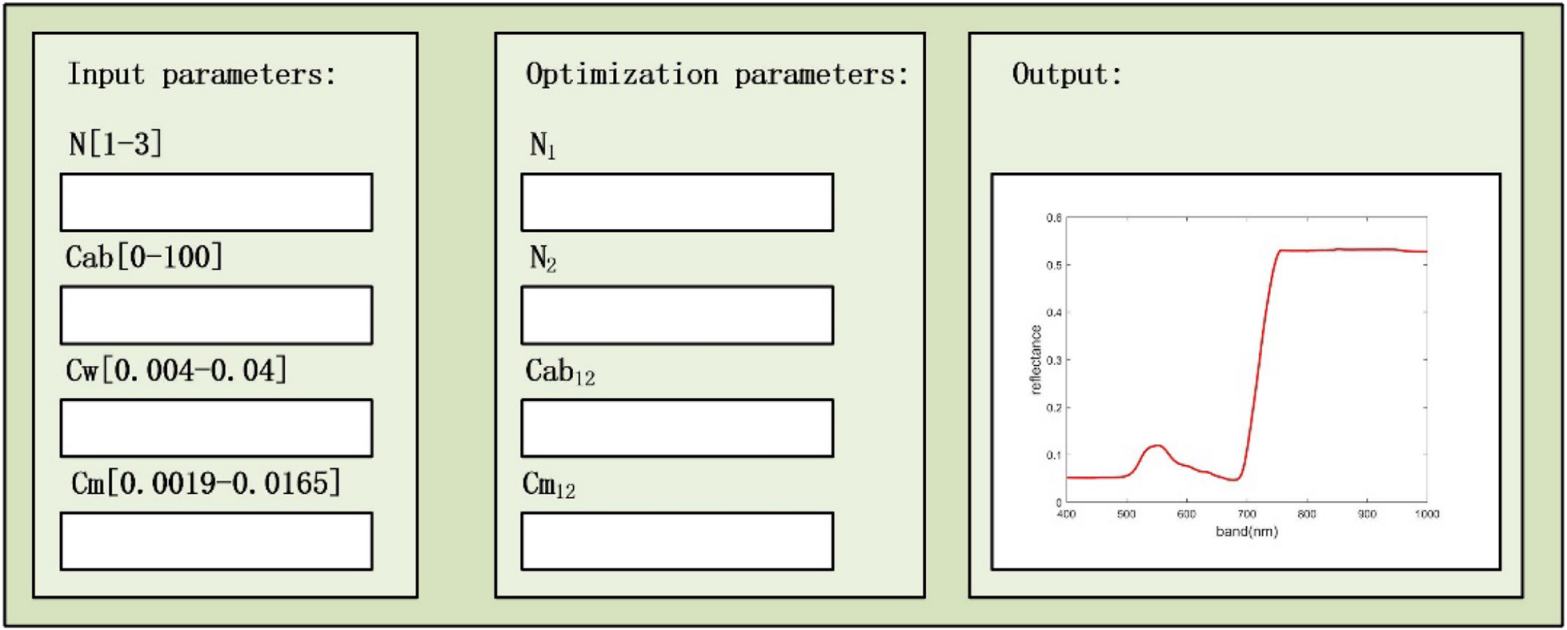
Interface diagram of the RPIOSL model

This paper adopts the concept of established the PIOSL model to presume rice leaves as two layers. The parameters of the model are defined using the NSGA -III algorithm, and the RPIOSL radiation transfer model for rice leaves is constructed. Figure 4 is the interface diagram of the RPIOSL model. The RPIOSL model has four input parameters, which involve N, Cab, Cw, and Cm. The optimized parameters include ${N}_{1}$、${N}_{2}$ of each layer, ${Cab}_{12}$ and ${Cm}_{12}$ of chlorophyll content. The construction principle of the rice radiative transfer model based on RPIOSL is introduced below.

### Effect of light on blade surface

A beam of natural light with a light intensity of 1 is emitted from the surface of the blade to the blade. Assuming that the surface of rice leaves is uniform and rough, Lambert reflection occurs on the surface of the leaves, and some light passes through the upper surface of the leaves and enters the inside of the leaves. Ignoring the absorption of light on the blade surface, and the expressions of transmissivity and reflectivity are obtained:

9 $$t_{1} \, = \,1\, - \,r_{1}$$

In the same way, the reflected light and transmitted light on the lower surface of the blade have the same relationship.

10 $$t_{2} \, = \,1\, - \,r_{2}$$

The values of T1 and T2 were derived by stern et al. according to the Fresnel equations Frank [11].

When the light passes through the surface of the blade, it will hit the 2π space outside the lower surface of the blade. The average transmittance ${t}_{\alpha v}(\alpha ,1,n)$ was calculated by Allen [2] when the incident angle is any angle.

Stern gives ${t}_{\alpha v}(\alpha ,1,n)$ and ${t}_{\alpha v}(\alpha ,n,1)$ relationship between:${t}_{\alpha v}\left(\alpha ,n,1\right)={n}^{-2}{t}_{\alpha v}(\alpha ,1,n)$

When the incident angleαWhen the same, ${t}_{\alpha v}\left(90,n,1\right)={n}^{-2}{t}_{\alpha v}(\mathrm{90,1},n)$ is substituted into formula (8)

$${t}_{1}={t}_{\alpha v}\left(\alpha ,1,n\right)$$,thus,$${r}_{1}=1-{t}_{\alpha v}\left(\alpha ,1,n\right)$$

### Stratification of the optical properties of rice leaves

In this paper, rice leaves are assumed to be two optical characteristic layers with structural parameter ${N}_{i}(i=\mathrm{1,2})$ and transmission coefficient ${\tau }_{i}({\text{i}}=\mathrm{1,2})$, and the specific absorption coefficient of absorbed substances in each layer is ${k}_{i}({\text{i}}=\mathrm{1,2})$ respectively.

The transmissivity of light passing through two layers is

11 $$\tau_{{i\left( {i = 1,2} \right)}} \, = \,Total\,transmitted\,energy/Total\,incident\,energy\, = \,\left( {1\, - \,k_{i} } \right)exp\left( { - k_{i} } \right)\, + \,k_{i}^{2} \mathop \smallint \limits_{{k_{i} }}^{\infty } t^{ - 1} exp^{ - 1} dt$$

where,$${k}_{i}\left(\lambda \right)=\sum {K}_{i}\left(\uplambda \right){C}_{i}/{N}_{i}$$

${C}_{i}$ and ${K}_{i}$ are the content of light-absorbing material and the corresponding specific absorption coefficient in the leaves, respectively In this paper, the PIOSL model is used to simulate the radiation transfer of rice leaves. The proportion of equivalent water thickness is close to 1 when the RPIOSL parameters are optimized by the NSGA—III algorithm. We simulated the reflectivity of rice leaves by stratifying the structural parameters, chlorophyll and dry matter of rice leaves and used the Cw as the input parameter of the upper layer of the leaves, this makes the optimization parameters of the model less and less time-consuming.

The light absorption coefficients of the two layers inside the blade are determined by Eqs. (11) and (12), respectively:

12 $$k_{1} \,\left( \lambda \right)\, = \,\sum K_{i} \,\left( \lambda \right)\,\left( {Cab_{12} \, * \,Cab\, + \,Cw\, + \,Cm_{12} \, * \,Cm} \right)/N_{1}$$

13 $$k_{2} \,\left( \lambda \right)\, = \,\sum K_{i} \,\left( \lambda \right)\,\left( {(1\, - \,Cab_{12} } \right)\, * \,Cab\, + \,\left( {1\, - \,Cm_{12} } \right)\, * \,Cm)/N_{2}$$

The absorption coefficient ${k}_{1}\left(\lambda \right)$ and ${k}_{2}\left(\lambda \right)$ is of each layer vary depending on the material content. ${K}_{i}\left(\lambda \right)$ is the specific absorption coefficient of the material in the blade that absorbs light in each band.

The parameter pairs of RPIOSL and PIOSL are shown in Table 2:

**Table 2:** Comparison of the parameters of the PIOSL and RPIOSL model

Mode;	Input parameter	Optimization parameter	Parameter interpretation	Function
PIOSL	N、Cab、Cw、Cm	${N}_{1}$、${N}_{2}$、${Cab}_{12}$、${Cw}_{12}$、${Cm}_{12}$、	${N}_{1}$, ${N}_{2}$ are the structural parameters of the first and second layers of leaves. The proportion of chlorophyll, water and dry matter content in the first and second layers of leaves are represented by ${Cab}_{12}$、${Cw}_{12}$、${Cm}_{12}$,respectively. Cab、Cw、Cm represent the total content of chlorophyll, water and dry matter in leaves, respectively	The biochemical parameters of various crops were output, and the Cab, Cw and Cm in the two layers of leaves were stratified, and the reflectivity of leaves was accurately simulated
RPIOSL	N、Cab、Cw、Cm	${N}_{1}$、${N}_{2}$、${Cab}_{12}$、${Cm}_{12}$	${N}_{1}$、${N}_{2}$ are the structural parameters of the first and second layers of leaves,${Cab}_{12}$、${Cm}_{12}$ are the proportion of Cab and Cm in the each layers of leaves, and Cab、Cw、Cm are the total content of chlorophyll, water and dry matter in leaves	The chlorophyll, water and dry matter contents of rice leaves were input, and the simulated reflectance of rice leaves was calculated by layering the Cab and Cm in the leaves. Compared with the PIOSL model, the parameters of ${Cw}_{12}$ were reduced, the calculation amount of the model was reduced, and the running time of the model was saved

### RPIOSL reflectivity calculation

**Fig. 5:**
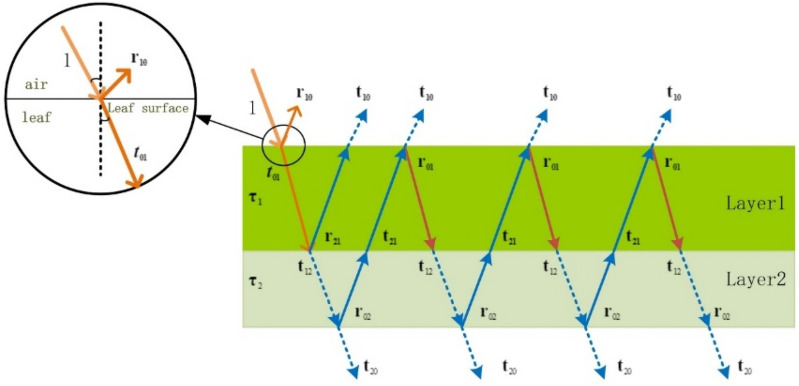
Light transmission process of the RPIOSL model

Figure 5 shows the radiation transmission process of a beam of natural light inside a rice leaf in the RPIOSL model. It is assumed that the upper surface of the blade receives a beam of natural light with energy of 1. Because the surface of the blade does not absorb the light, some of the light incident on the upper surface of the blade is reflected and the rest is transmitted. Let the reflected energy be ${r}_{10}$ and the transmitted energy be ${t}_{01}$,and the light passing through the blade surface enters the blade to participate in transmission. First, it will be absorbed by elements or other substances in the first layer of the blade, and the rest will be reflected to the first layer of the blade or transmitted to the second layer of the blade. Let the reflected energy of this part be R1 and the transmitted energy be T1. T1 enters the second layer of the blade to participate in transmission, and is absorbed by elements or other substances in the second layer. The rest will be reflected to the first layer of the blade or transmitted to the lower surface of the blade. Let the reflectivity be R2 and the transmittance be T2. The reflectivity interacts with the upper layer of the blade until the energy of light decreases to 0.

The reflectivity of the upper surface of the blade is

14 $${\text{R}}1\, = \,r_{10} \, + \,t_{01} \tau_{1} r_{21} \tau_{1} t_{10}$$

The transmissivity of light after absorption by the first layer is

15 $${\text{T}}1\, = \,\frac{{t_{12} \,\left( {a\, - \,a^{ - 1} } \right)}}{{ab^{N1 - 1} \, - \,a^{ - 1} b^{1 - N1} \, - \,\left( {b^{N1 - 1} \, - \,b^{1 - N1} } \right)R_{90} \left( 1 \right)}}$$

where,

$${\text{a}}=(1+{R}_{90}^{2}\left(1\right)-{r}_{90}^{2}\left(1\right)+\delta )/2{R}_{90}(1)$$

$${\text{b}}=(1+{R}_{90}^{2}\left(1\right)-{r}_{90}^{2}\left(1\right)-\delta )/2{R}_{90}(1)$$

$$\updelta =\sqrt{{({R}_{90}^{2}\left(1\right)-{T}_{90}^{2}\left(1\right)-1)}^{2}-4{T}_{90}^{2}\left(1\right)}$$ or $$\sqrt{{({T}_{90}^{2}\left(1\right)-{R}_{90}^{2}\left(1\right)-1)}^{2}-4{R}_{90}^{2}\left(1\right)}$$

Part of the energy reflected by T1 after absorption by the second layer is

16 $${\text{R}}2\, = \,t_{12} \, \cdot \,\tau_{2} r_{02} \tau_{2}$$

The transmittance of T1 in the lower layer of the blade is

17 $${\text{T}}2\, = \,t_{12} \, \cdot \,\tau_{2} t_{20}$$

Therefore, the total reflectivity can be expressed as follows:

18 $${\text{R}}\, = \,{\text{R}}1\, + \,{\text{R}}2t_{21} \tau_{1} t_{10} \, + \,{\text{R}}2t_{21} \tau_{1} t_{10} \tau_{1} t_{12} \tau_{2} r_{02} \tau_{2} t_{21} \tau_{1} t_{`10}$$

The total transmittance can be rewritten as:

19 $${\text{T}}\, = \,{\text{T}}2\, + \,t_{12} \tau_{2} r_{02} \tau_{2} t_{21} \tau_{1} r_{01} \tau_{1} t_{12} \tau_{2} t_{20} \, + \,{\text{T}}1\tau_{2} r_{02} \tau_{2} t_{21} \tau_{1} r_{01} \tau_{1} t_{12} \tau_{2} r_{02} \tau_{2} t_{21} \tau_{1} r_{01} \tau_{1} t_{12} \tau_{2} t_{20} \, + \, \ldots$$

Substitute R1, R2, T1 and T2 into R and t to get

20 $${\text{R}}\, = \,r_{10} \, + \,t_{01} \tau_{1} r_{21} \tau_{1} t_{10} \, + \,t_{12} \tau_{2} r_{02} \tau_{2} t_{21} \tau_{1} t_{10} \, + \,t_{12} \tau_{2} r_{02} \tau_{2} t_{21} \tau_{1} r_{01} \tau_{1} t_{12} \tau_{2} r_{02} \tau_{2} t_{21} \tau_{1} t_{`10} \, = \,r_{10} \, + \,t_{01} \tau_{1} r_{21} \tau_{1} t_{10} \, + \,\frac{{t_{12} \tau_{2} r_{02} \tau_{2} t_{21} \tau_{1} t_{10} }}{{1\, - \,r_{01} \tau_{1} t_{12} \tau_{2} r_{02} \tau_{2} t_{21} \tau_{1} }}$$

21 $${\text{T}}\, = \,t_{12} \tau_{2} t_{20} \, + \,t_{12} \tau_{2} r_{02} \tau_{2} t_{21} \tau_{1} r_{01} \tau_{1} t_{12} \tau_{2} t_{20} \, + \,t_{12} \tau_{2} r_{02} \tau_{2} t_{21} \tau_{1} r_{01} \tau_{1} t_{12} \tau_{2} r_{02} \tau_{2} t_{21} \tau_{1} r_{01} \tau_{1} t_{12} \tau_{2} t_{20} \, + \, \ldots \, = \,\frac{{t_{12} \tau_{2} t_{20} }}{{1\, - \,r_{02} \tau_{2} t_{21} \tau_{1} r_{01} \tau_{1} t_{12} \tau_{2} }}$$

${\tau }_{1}$ and ${\tau }_{2}$ are the transmittance produced by the absorption of light in the upper and lower layers of the blade when it passes through the upper and lower layers of the blade. ${r}_{ij}$、${t}_{ij}$(i,j) represent the reflectivity and transmittance from medium i to medium j.

## Result

### Optimization of the RPIOSL parameter

**Fig. 6:**
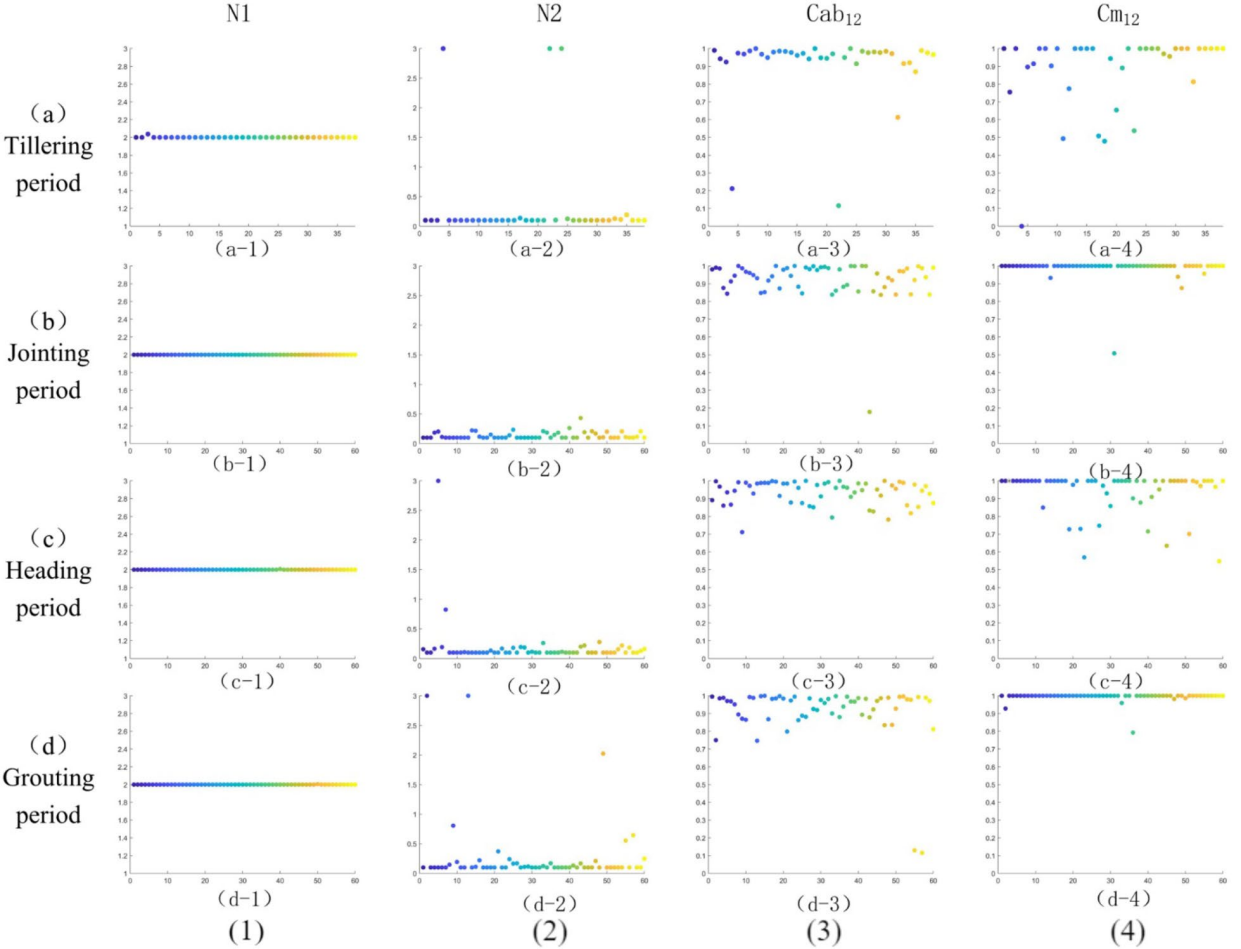
Optimization results of parameters in each period

In order to stratify the contents of the absorbent substances in each layers after internal stratification of rice leaves, the NSGA-III. algorithm was used to define N1 and N2, ${Cab}_{12}$ and ${{\text{Cm}}}_{12}$ of RPIOSL model. In order to distinguish the hierarchical structure of rice leaves with different growth cycles, we optimized the parameters at different stages of growth and development. Figure 6 shows the parameter optimization results of rice at the tillering period, jointing period, heading period and grouting period.

From Fig. 6, it can be seen that the optimization results of rice leaf parameters are consistent at tillering period, jointing period, heading period, and grouting period. Among them, the consistency of the structural parameters of the first floor is good, and most of the sample points are around 2. The value of the structural parameter N2 was close to 0–0.5, the proportion of chlorophyll content was distributed mainly in 0.7–1, and the consistency of ${Cab}_{12}$ in rice tillering period was good. Table 3 shows the calculated data of the structural parameters and the classification of rice material content in each period. The average proportion of ${Cab}_{12}$ in the four growth periods of rice can be found in Table 3, which are 0.9130, 0.9245, 0.9346 and 0.9124. Therefore, the Cab of rice leaves tends to concentrate in the first layer. Figure 6a-4, b-4, c-4 and d-4 reveal that ${Cm}_{12}$ is mainly distributed between 0.5 and 1. Therefore, the dry matter of rice leaves is mainly distributed in the first layer at each stage of rice. It can be observed that the average ${Cm}_{12}$ of rice leaves in four different periods is 0.8813, 0.9869, 0.9422, and 0.8841, respectively. The parameter optimization results for the entire period are displayed in Table 3, with average values of N1, N2, ${Cab}_{12}$, and ${Cm}_{12}$ equal to 2.0002, 0.2233, 0.9220, and 0.9582, respectively. Overall, although there are some differences in the stratification of rice leaves at different periods of growth and development, the proportion of stratification of structural parameters and rice leaves' material content remains consistent.

**Table 3:** Optimization results of RPIOSL model parameters

	Parameter	N1	N2	${Cab}_{12}$	${Cm}_{12}$
Tillering period	Min	2	0.1	0.1158	0
	Max	2.0373	3	1	1
	Mean	2.0010	0.3342	0.9130	0.8813
	Variance	0.00004	0.6258	0.0362	0.0476
Jointing period	Min	2	0.1	0.1780	0.5080
	Max	2.00001	0.4312	1	1
	Mean	2.0000001	0.1351	0.9245	0.9869
	Variance	1.2944E-12	0.0037	0.0130	0.0044
Heading period	Min	2	0.1	0.7115	0.5477
	Max	2.0068	3	1	1
	Mean	2.0001	0.1858	0.9346	0.9422
	Variance	7.67106E-07	0.1466	0.0044	0.0134
Grouting period	Min	2	0.1	0.1157	0.7927
	Max	2.0052	3	1	1
	Mean	2.0001	0.2788	0.9124	0.8841
	Variance	4.50976E-07	0.3352	0.0261	0.0010
All periods	Min	2	0.1	0.1157	0
	Max	2.0373	3	1	1
	Mean	2.0002	0.2233	0.9220	0.9582
	Variance	6.70453E0-06	0.2443	0.0181	0.0148

**Fig. 7:**
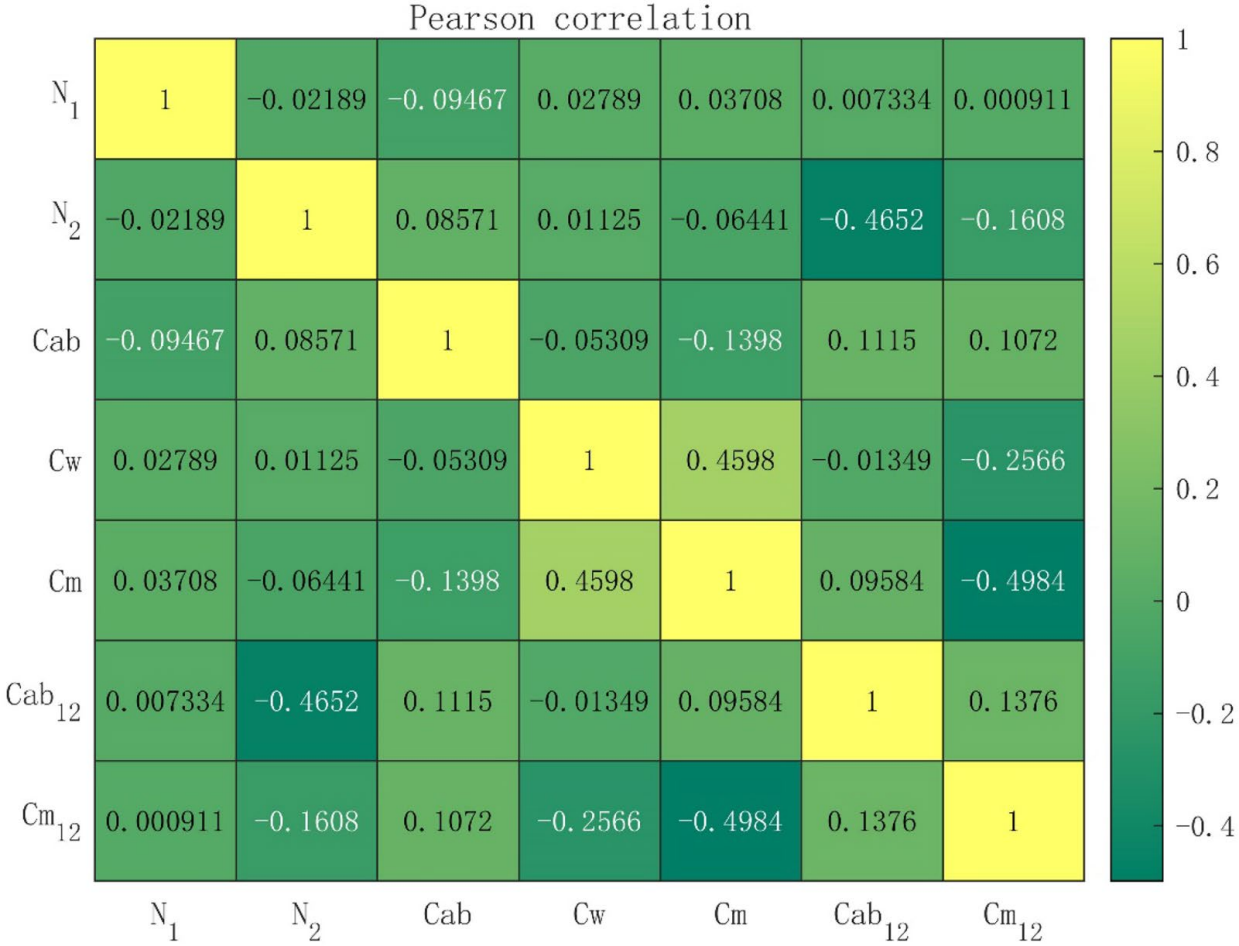
Pearson correlation analysis results

As the input parameters of RPIOSL model include the measured parameters including chlorophyll content, equivalent water thickness and dry matter content, the simulation parameters include the structural parameters of the first and second layers of leaves, the ratio of chlorophyll content in the first and second layers of leaves and the ratio of dry matter content in the first and second layers of leaves. Therefore, we have carried out correlation analysis on the measured parameters and simulated parameters of RPIOSL model. Figure 7 is the Pearson correlation analysis result. From the results of correlation analysis, it can be seen that the structural parameters of the second layer are negatively correlated with the proportion of chlorophyll in leaves, and the correlation coefficient is −0.4652; There is a negative correlation between dry matter content and the proportion of dry matter content, and the correlation coefficient is −0. 4984; There is a positive correlation between equivalent water thickness and dry matter content, and the correlation coefficient is 0.4598.

### Simulated spectral comparison

**Fig. 8:**
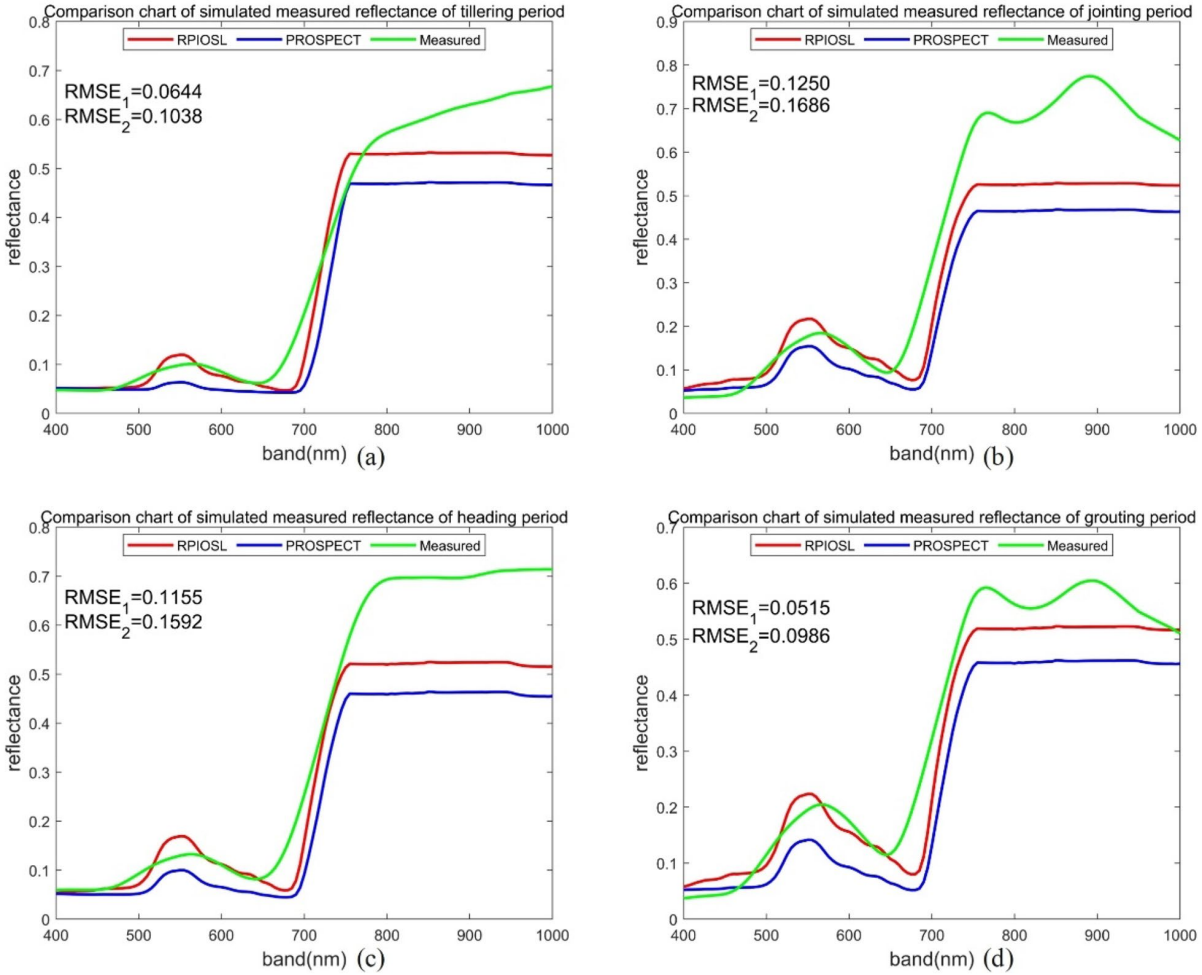
RPIOSL model simulation spectral effect diagram (${RMSE}_{1}$ and ${RMSE}_{2}$ are the RMSE of the simulated and measured spectra of the RPIOSL model and PROSPECT model at 400–1000 nm respectively)

In this paper, we simulated the reflectance spectra of 218 samples using the RPIOSL and PROSPECT models with input of measured Cab, Cw, and Cm. The proportion of material content in each layer was optimized using the NSGA—III optimization algorithm. Figure 8 shows the comparison of simulated spectra of rice at tillering stage, jointing stage, heading stage and filling stage by RPIOSL model and PROSPECT model.

Figure 8 demonstrates that the simulation effectiveness of leaf reflectivity using the RPIOSL model outperforms that of the PROSPECT model. Across the four samples visualized in Fig. 8, the RMSE of the simulated spectrum and the measured spectrum through the RPIOSL model were 0.0644, 0.1250, 0.1155 and 0.0515, respectively, which were 0.0394, 0.0436, 0.0437 and 0.0471 lower than those of the PROSPECT. The simulated spectrum of the RPIOSL model is closer to the real spectrum in shape, particularly in the visible light band of 480–650 nm. The simulated spectrum of the RPIOSL model in the 750–1000 nm band is lower than the actual value, but it is higher than that of the PROSPECT. The simulation effect of the strong absorption peak at 690 nm is poor, which may be due to the lack of calibration of the specific absorption coefficient curve of chlorophyll. From the four growth cycles of rice, the simulation effect of RPIOSL on the tillering and grouting periods of rice is better. At the jointing period and heading period, the simulation value of RPIOSL model in the near-infrared band is lower, but it is better than that of the PROSPECT model.

It can be seen from the Fig. 8 that the simulation effect of RPIOSL model for 650–1000 nm spectrum is really not ideal. I think the following reasons limit the simulation effect of RPIOSL model for 650–1000 nm reflectivity spectrum. First, the reflectivity of 650–1000 nm is mainly affected by water and dry matter. It may be that the measurement results of water or dry matter in experimental data are not accurate enough, which leads to the decline of simulation accuracy of RPIOSL model. Secondly, the specific absorption coefficients of water and dry matter were not calibrated when constructing the RPIOSL model. In fact, there may be some differences between the internal structure of rice leaves and other leaves, so it is impossible to simply use the absorption coefficient curves of various crops instead of the absorption coefficient curves of rice leaves, which is also one of the reasons for the decline of simulation accuracy of RPIOSL model. Thirdly, the measured spectra may be affected by the light source, resulting in some noise fluctuations, which causes the measured spectra of 650–1000 nm to be on the high side. There are many reasons for the degradation of the simulation accuracy of 650–1000 nm spectra, and further research is needed to improve the simulation accuracy of this band.

### Segmented comparison of spectra

In this paper, the RPIOSL and PROSPECT models were used to input N, Cab, Cw, and Cm of leaves, obtaining reflectance spectra of leaves at 400–1000 nm. This study utilized the RPIOSL and PROSPECT models to input N, Cab, Cw, and Cm of leaves, obtaining reflectance spectra of leaves at 400–1000 nm. As shown in Fig. 8, the RPIOSL model displays a superior spectral simulation effect on rice leaf reflectivity. The RMSE at the four growth periods in Fig. 8 were 0.0644, 0.1250, 0.1155, and 0.0515, respectively, which were 0.0394, 0.0436, 0.0437, and 0.0471 lower than PROSPECT model. This indicates that the hypothesis of blade division into two layers with distinct optical properties is valid. By optimizing the element content ratio of each layer, an accurate simulation of leaf reflectance spectra can be achieved.

**Fig. 9:**
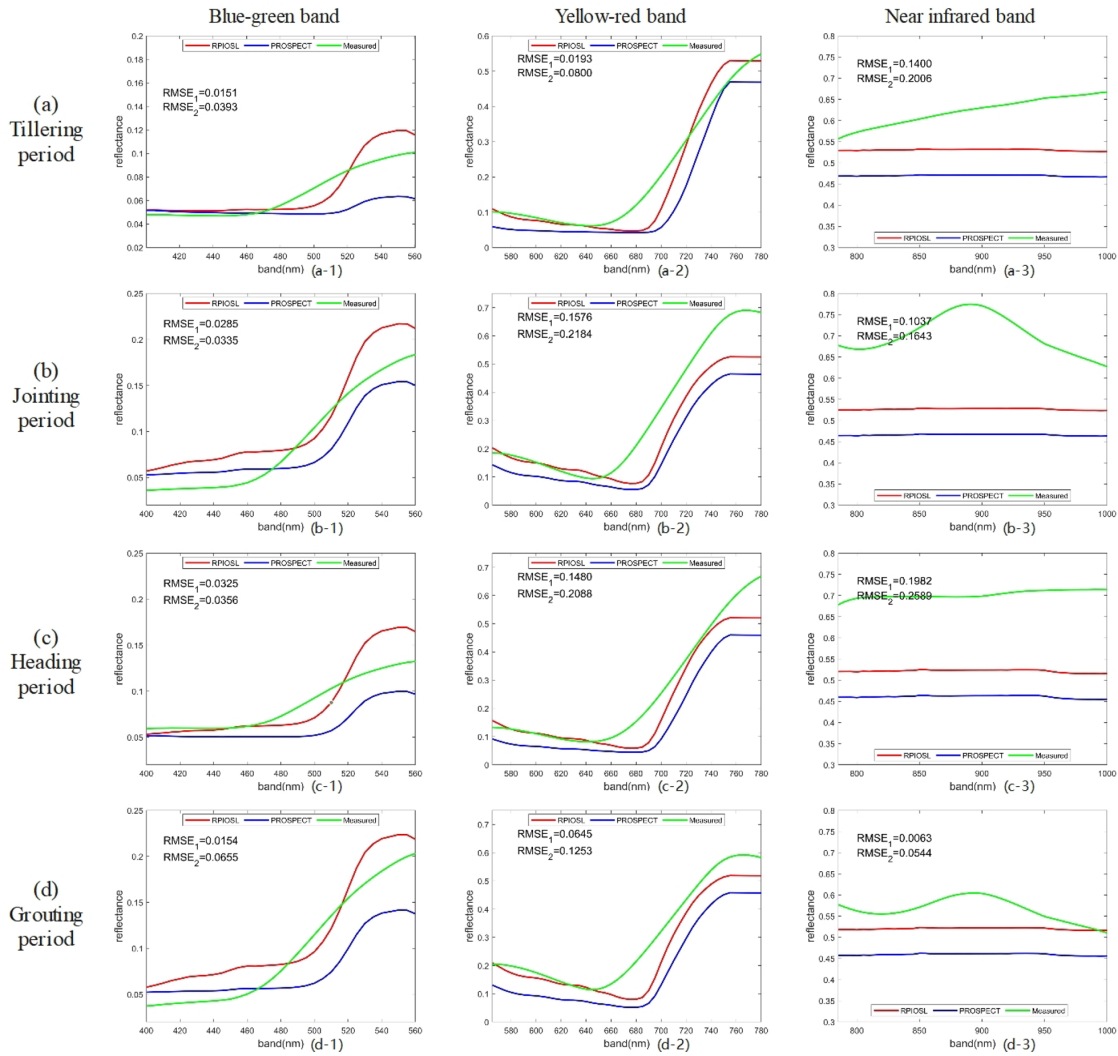
Comparison of the simulated spectrum and the measured spectrum at different bands in RPIOSL model and the PROSPECT

**Fig. 10:**
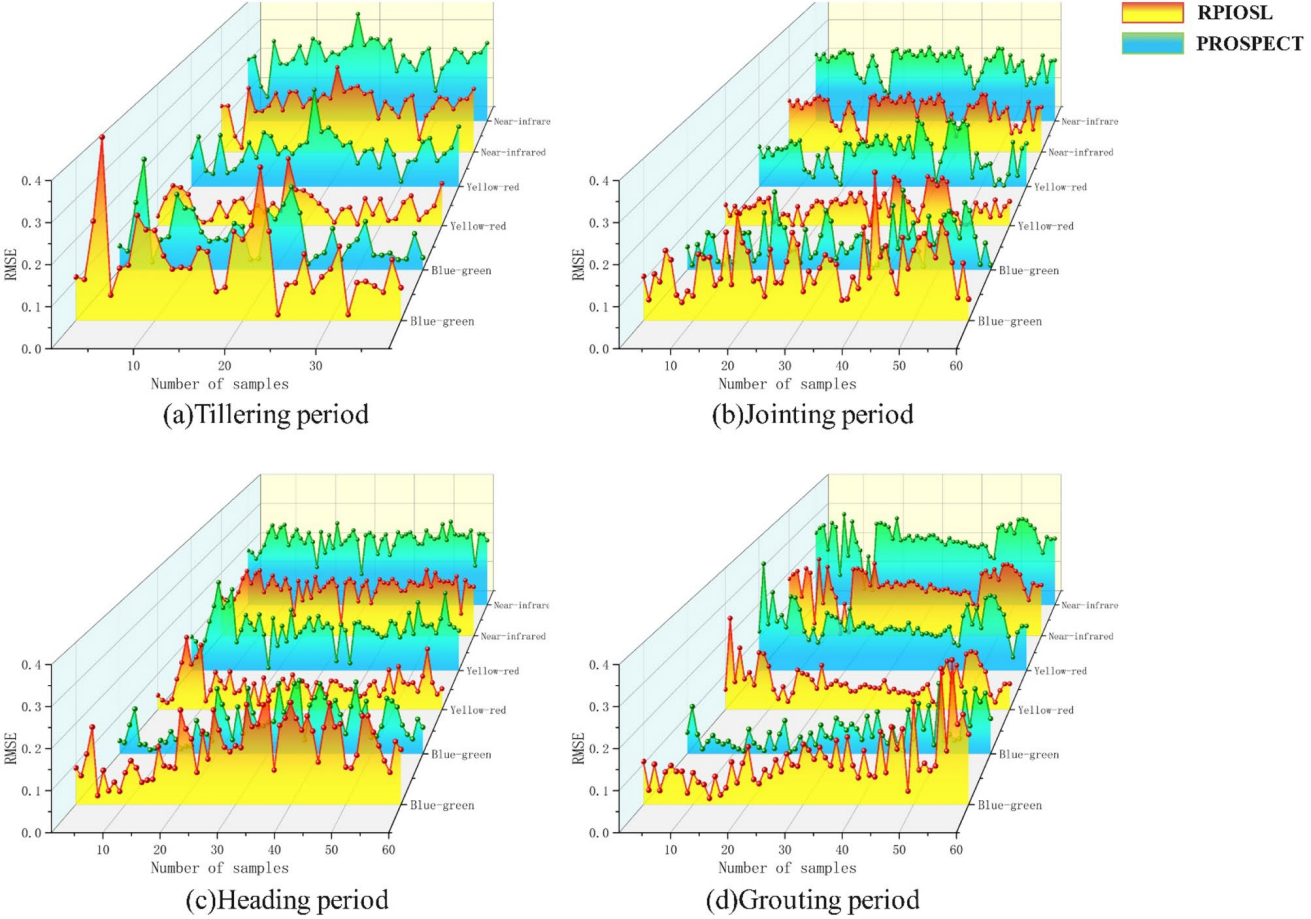
Comparison of RMSE of RPIOSL model and the PROSPECT model at different wave bands at different periods of rice

To compare the differences between the simulated spectra of the RPIOSL and PROSPECT models with respect to stratification of substances inside leaves in different bands, the RPIOSL simulated spectra were mapped in the blue-green light band (400–560 nm), yellow–red light band (560–780 nm), and near-infrared band (780–1000 nm). The difference between the predicted spectra and the measured spectra is displayed in Fig. 9. According to the RMSE results of statistical simulation of the RPIOSL and PROSPECT models presented in Table 4, in the blue-green light band, the RPIOSL model has the highest accuracy during the grouting period, with an average RMSE of 0.1099, but the RMSE for all four periods are all relatively high, exceeding PROSPECT model. Figure 10 shows the distribution of RMSE values for measured simulated spectral differences between the RPIOSL and PROSPECT models during different growth periods and bands of rice. It can be observed from Fig. 10 that the RMSE of the RPIOSL model is lower than that of the PROSPECT model in the yellow red band and near-infrared band, except for the RMSE in the blue-green band, which is higher than PROSPECT. The simulation performance of the RPIOSL model is best in the yellow–red light band, with mean RMSE values of 0.0584, 0.0576, 0.0724, and 0.0820 at the tillering, jointing, heading, and grouting periods, which are 0.0545, 0.0467, 0.0589, and 0.0603 lower than PROSPECT, respectively.

**Table 4:** RMSE statistical results of rice spectral simulation at different periods and bands by the RPIOSL model and the PROSPECT model

			Min	Max	Mean
Tillering period	Blue-green band	RPIOSL	0.0148	0.4526	0.1497
		PROSPECT	0.0159	0.2925	0.0942
	Yellow–red band	RPIOSL	0.0035	0.1894	0.0584
		PROSPECT	0.0149	0.2907	0.1129
	Near infrared band	RPIOSL	0.0130	0.2691	0.1553
		PROSPECT	0.0804	0.3591	0.2204
Jointing period	Blue-green band	RPIOSL	0.0449	0.3658	0.1369
		PROSPECT	0.0044	0.2106	0.0793
	Yellow–red band	RPIOSL	0.0006	0.1386	0.0576
		PROSPECT	0.0030	0.1993	0.1043
	Near infrared band	RPIOSL	0.0340	0.1859	0.1341
		PROSPECT	0.0946	0.2464	0.1953
Heading period	Blue-green band	RPIOSL	0.0222	0.2566	0.1444
		PROSPECT	0.0105	0.1943	0.0889
	Yellow–red band	RPIOSL	0.0124	0.2041	0.0724
		PROSPECT	0.0080	0.2648	0.1313
	Near infrared band	RPIOSL	0.0392	0.2096	0.1564
		PROSPECT	0.1032	0.2793	0.2193
Grouting period	Blue-green band	RPIOSL	0.0154	0.3568	0.1099
		PROSPECT	0.0055	0.1866	0.0604
	Yellow–red band	RPIOSL	0.1041	0.2578	0.0820
		PROSPECT	0.0402	0.3204	0.1423
	Near infrared band	RPIOSL	0.0063	0.2435	0.1620
		PROSPECT	0.0494	0.3041	0.2222

It can be seen from (a-1), (b-1), (c-1) and (d-1) in Fig. 9 that in the blue light band of 400-490 nm, the simulation effect of the RPIOSL model at tillering and heading periods of rice is better than that of the PROSPECT model, and the simulated spectrum at jointing and grouting periods is much lower than the measured value. In the 490–560 green light band, the simulation effect of the RPIOSL and the PROSPECT is general. The simulation value of the former is higher for the reflectance peak near 540 nm, while that of the latter is lower. Research has shown that in the visible light band, various pigments are the main factors controlling the spectral response of plants, with chlorophyll playing the most important role. Chlorophyll absorbs most of the incident energy in two spectral bands with central wavelengths of 450 nm (blue) and 650 nm (red), respectively. In between the two chlorophyll absorption bands, a reflection peak is formed at 540 nm due to the small absorption effect [25]. Because the RPIOSL model includes carotenoids and anthocyanins in chlorophyll as an input parameter, it affects the simulation of the chlorophyll strong absorption peak to some extent.

In the yellow and red light band, the simulation effect of the RPIOSL model on the tillering period and the jointing period was better, and the mean RMSE was 0.0584 and 0.0576, respectively. However, it can be observed from Fig. 9a-2, b-2, c-2 and d-2 that the simulation effect of the RPIOSL model on the strong absorption peak at 650 nm is not ideal. This may be due to the fact that the chlorophyll of the RPIOSL model parameter contains carotenoids, anthocyanins, and other elements with absorption characteristics. Therefore, in future research, introducing new input parameters such as carotenoids or anthocyanins and optimizing the chlorophyll characteristic absorption coefficient of the RPIOSL model can increase the simulation effect of the RPIOSL model in the visible light band. In the near-infrared band, both the simulated spectra of the RPIOSL and PROSPECT models underestimate the measured values, but the RPIOSL model's performance is superior to that of the PROSPECT model. The RMSE at the tillering, jointing, heading, and booting periods are 0.1400, 0.1037, 0.1982, and 0.0063, respectively, which are 0.0605, 0.0606, 0.0607, and 0.0481 lower than the PROSPECT model at the same periods, respectively. Spectral characteristics in the near-infrared band are mainly affected by water and dry matter [23, 26]. Therefore, the key to improving the near-infrared spectral simulation effect is to calibrate the absorption coefficient of water and dry matter in the RPIOSL model.

### Comparison of the RMSE

**Fig. 11:**
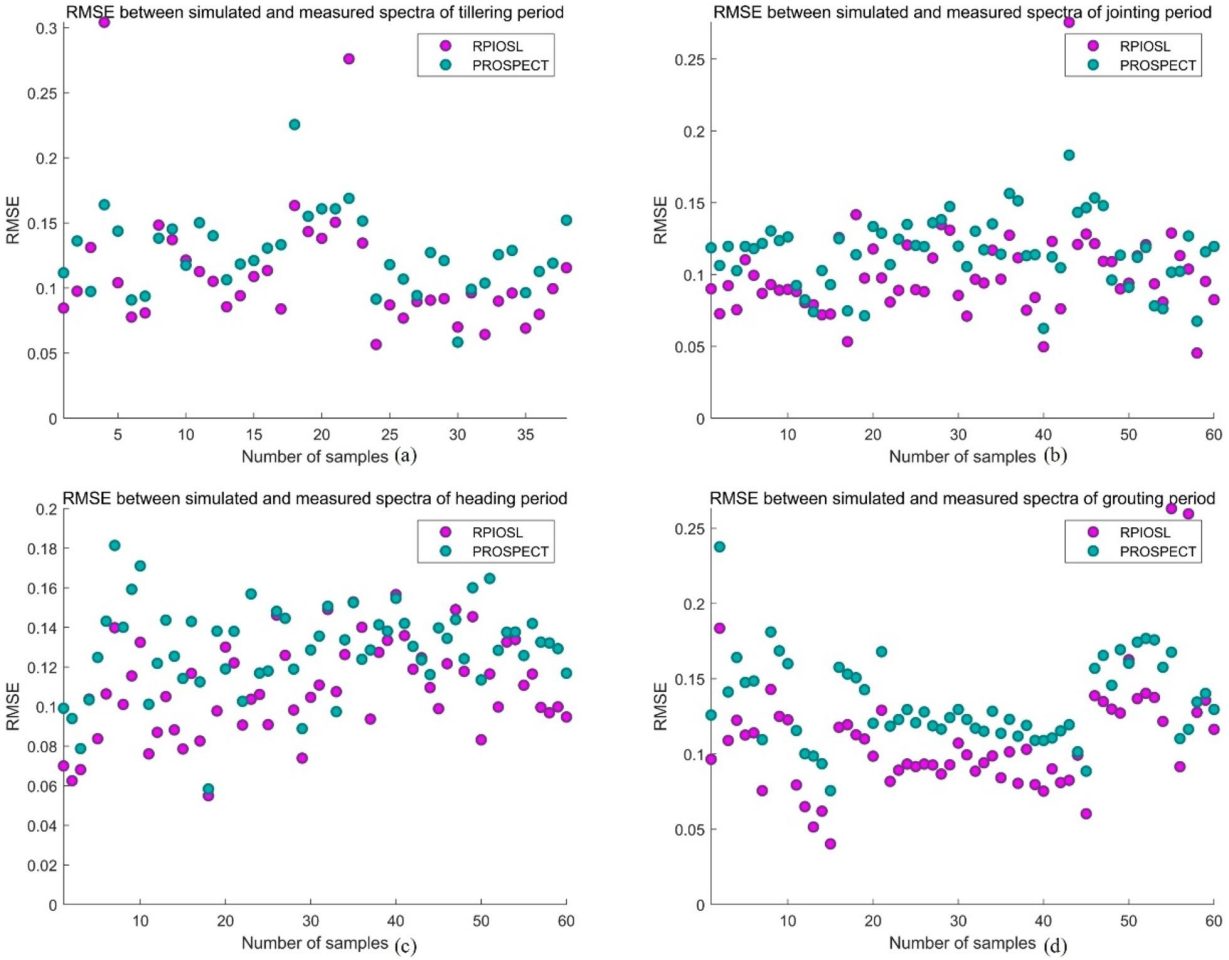
Variation of the RMSE of RPIOSL and PROSPECT with samples

**Fig. 12:**
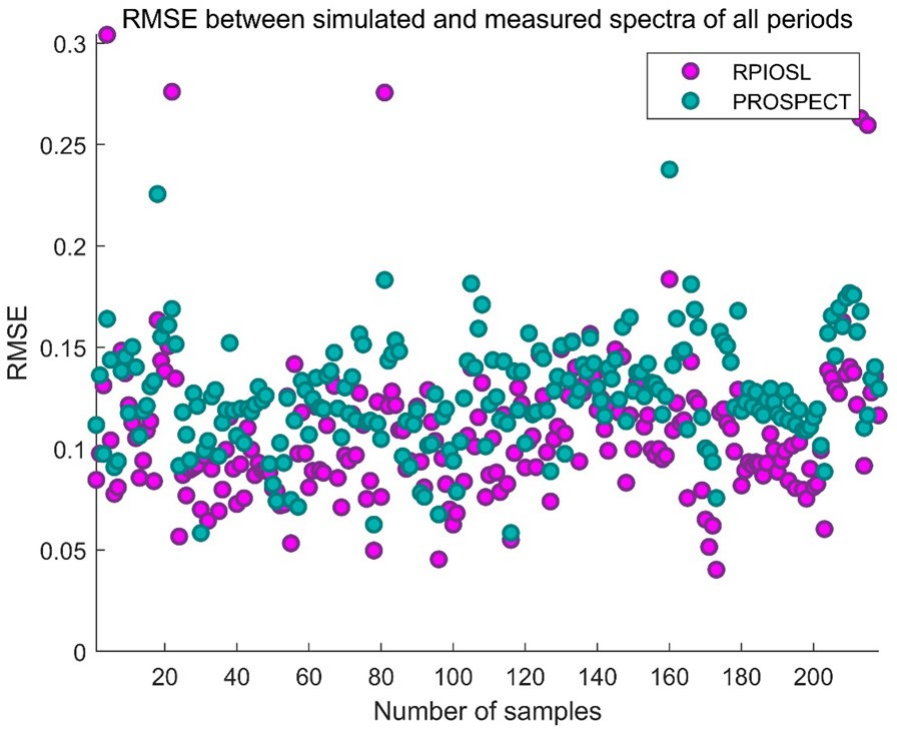
RMSE in the entire growth cycle

In this paper, the efficiency of RPIOSL model and PRSPECT model is evaluated by calculating the sum of errors between simulated spectra and measured spectra in each band. Figure 11 demonstrates the change of RMSE between the simulated and measured spectra of the RPIOSL model and the PROSPECT model with rice samples from each growth period. It can be seen from Fig. 11 that the RMSE of the RPIOSL model at each growth period of rice is lower than that of PROSPECT. Table 5 displays the RMSE results of the RPIOSL model and PROSPECT model in each growth period. It can be observed from Table 5 that the mean RMSE of the RPIOSL model in the tillering stage, jointing stage, heading stage, and grain grouting stage were 0.1124, 0.1001, 0.1094 and 0.1094 respectively, which were 0.0144, 0.0155, 0.02 and 0.0248 lower than those of the PROSPECT model. Over the entire rice growth cycle, the RMSE of the RPIOSL model was 0.0191 lower than that of PROSPECT. Figure 12 compares the RMSE of the RPIOSL model and the PROSPECT model during the entire rice growth period. It can be seen that the RMSE of the RPIOSL model at all rice growth stages is lower than that of the PROSPECT model.

**Table 5:** RMSE statistical results of the RPIOSL and PROSPECT models

		RPIOSL	PROSPECT
Tillering period	Min	0.0566	0.0584
	Max	0.3041	0.2256
	Mean	0.1124	0.1268
	Variance	0.0025	0.0009
Jointing period	Min	0.0533	0.0713
	Max	0.2756	0.1832
	Mean	0.1001	0.1156
	Variance	0.0010	0.0006
Heading period	Min	0.0550	0.0584
	Max	0.1566	0.1814
	Mean	0.1094	0.1294
	Variance	0.0006	0.0005
Grouting period	Min	0.0403	0.0756
	Max	0.2630	0.2377
	Mean	0.1094	0.1342
	Variance	0.0015	0.0008
All periods	Min	0.0403	0.0584
	Max	0.3041	0.2377
	Mean	0.1074	0.1265
	Variance	0.0013	0.0007

## Discussion

### Layered idea of the RPIOSL model

After years of development, the input parameters and assumptions of the PROSPECT model have undergone significant changes. Beginning with the original PROSPECT-3 model with only three input parameters, it has evolved into the PROSPECT-PRO model that includes carotenoids, anthocyanins, nitrogen-containing proteins, and CBC parameters. From the initial model of a blade as a superposition of n-layer flat plates with the same properties, we have the PIOSL model of assuming the blade as a multilayer optical property of different layers. The leaf layering assumption is consistent with the actual physiological characteristics of leafy vegetation. The structural differences in rice leaves and the heterogeneity of biochemical parameters impact the photosynthetic efficiency of rice leaves. The relationship between gas exchange probability in leaves and leaf tissue structure of two rice varieties was studied by Xiong [30], indicating that changes in leaf function are related to changes in structure and chemistry, and that photosynthetic heterogeneity in leaves should be taken into account. In this paper, the energy transmission calculation of light in the layered structure of rice leaves was realized through the layered optical characteristics of rice leaves, and the material content of each layer after layered rice leaves was determined.

Whether it is at the tillering, jointing, heading, or booting period, it can be observed from Fig. 6a, b, the parameter optimization results for rice that the structural parameters of the first layer of the RPIOSL model’s are generally greater than those of its second layer. ${N}_{1}$ approaches 2, and only individual sample points of tillers have slight deviation.${N}_{2}$ tends to be 0–0.5, but the abnormal value of ${N}_{2}$ in individual sample points at each period of rice reaches 3, which is inconsistent with the theoretical distribution law. The parameter settings when using NSGA—III optimization are as follows:

22 $$N_{1} \, = \,N\, * \,\left( {2\, + \,0.01\, * \,x\,\left( 1 \right)} \right)$$

23 $$N_{2} \, = \,N\, * \,\left( {0.1\, + \,2.9\, * \,x\,\left( 2 \right)} \right)$$

where, $$x\left(1\right),x(2)\in (\mathrm{0,1})$$。

Therefore, the abnormal value of the optimization of the structural parameter N2 may be the local optimal solution when using the NSGA—III algorithm. Here N1 and N2 characterize the structural complexity of leaves, indicating that after layering the optical properties of rice leaves, the structural complexity of the first layer of leaves is higher than that of the second layer. In fact, the physical quantity of structural parameters has no actual physical meaning. She can only calibrate its value by optimizing the parameters. It is based on conceptual quantities rather than measurable physiological traits. Theoretically, the structural parameters of leaves are related to the structural characteristics of leaves, such as the thickness of leaves, the distance between cells, including the number of airspace and the size of mesophyll cells. The structural parameters of leaves will also affect the photosynthetic efficiency of plants. Wider leaves can increase the chlorophyll content of leaves and improve the photosynthetic efficiency of plants; Longer leaves can increase the surface area of leaves and improve the absorption and utilization rate of light energy by plants. Therefore, the structural parameters of leaves are closely related to photosynthesis, and then affect the growth and development of plants. These structural characteristics of leaves are concentrated in structural parameter N, which was previously associated with physical properties of leaves, such as specific leaf area, which was obtained based on empirical relationship. The RPIOSL model constructed in this paper layered the structural parameters, and explored the role of structural parameters in each layer of the blade in more detail. In the RPIOSL model, N is used as a parameter to describe the structural characteristics of the blade as input to the model, and the simulation of the reflectivity of the blade is performed. As a parameter without practical physical meaning, the existence of N in the model leads to the instability of the model. Therefore, during the development of the RPIOSL model, we should focus on the relationship between structural parameters and actual blade parameters and look for physical quantities that can replace structural parameters. At the same time, the epidermal structure of rice leaves also has a certain impact on the transmission process of light radiation. The effect of 24-Epibrassinolide (EBR) effect of rice plants under simulated acid rain treatment(SAR) studied by Da Fonseca [6], and found that SAR increased the density of the trichomes, epidermal wax and the stomatal area, and improved the tolerance of rice plants. As shown in Fig. 3, the scanning electron microscope of the upper surface of rice leaves at various periods shows that the pores and bulges on the upper surface of rice leaves affect the surface roughness, which is inconsistent with the assumption of wave reflection on the leaf surface of the RPIOSL model. Therefore, in future research, it is also crucial to take into account the impact of epidermal structure features on the optical properties of rice leaves. I believe that the RPIOSL rice radiation transfer model with skin structure can accurately simulate the optical properties of rice leaves and further enhance the optimization results of the structural parameters.

When the chlorophyll and dry matter of the leaves were stratified, it was found that both chlorophyll and dry matter had a trend of more in the first layer. Due to the Cw proportion being close to 1 in the optimization process, this article does not present the stratification of the equivalent water thickness. To reduce the optimization time, we set ${Cw}_{12}$ of RPIOSL at 1, which has no significant impact on the accuracy of the model. It can be observed from Fig. 6c, d that the distribution of chlorophyll and dry matter content in rice is consistent at various growth periods. Among them, the proportion of chlorophyll to ${Cab}_{12}$ is more consistent in the tillering period of rice. According to Table 3, the average value of ${Cab}_{12}$ at the tillering period, jointing period, heading period and grouting period of rice is 0.9130, 0.9245, 0.9346 and 0.9124 respectively. This indicates that the Cab of rice leaves during the heading period is concentrated more in the first layer. The heading period is the alternate period of reproductive growth and vegetative growth of rice, and rice leaves need to improve photosynthetic efficiency obtain more nutrients in the heading period. For the proportion of dry matter content, ${Cw}_{12}$ also has the same trend of focusing on the first layer, and the consistency of ${Cw}_{12}$ is better than ${Cab}_{12}$. The mean values of ${Cw}_{12}$ in the tillering period, jointing period, heading period and grouting period were 0.8813, 0.9869, 0.9422 and 0.8841, respectively, and the variance was lower than 0.0476. In general, the chlorophyll and dry matter of the rice leaves tended to concentrate in the first layer, with an average of 0.9220 and 0.9582, respectively.

In addition to the elements considered by RPIOSL, leaf nitrogen plays an important regulatory role in photosynthesis and respiration of vegetation leaves [29]. However, it is very difficult to deduce the nitrogen content from massive spectral data without damage. The original PROSPECT model's chlorophyll absorption coefficient was swapped out for the equivalent N absorption coefficient, creating n-PROSPECT, which accurately simulated both leaf reflectance and nitrogen concentration by Yang [32]. In the future, we will consider adding nitrogen as an input parameter to the RPIOSL model to study the effect mechanism of nitrogen on the rice leaf spectrum. Although the RPIOSL model constructed in this paper can accurately simulate leaf reflectance, it is improved on the basis of the PIOSL model. The existing PROSPECT and its improved versions, such as PROSPECT-5 and PROSPECT-d, have separated carotenoids and anthocyanins from chlorophyll. The PIOSL model and RPIOSL did not stratify carotenoids and anthocyanins in the upper and lower layers of leaves, because the content of carotenoids and anthocyanins in the leaves of vegetation was low, so it was difficult to achieve an accurate division [3, 28]. In future research, the distribution of these low- content elements in leaves will be considered, including carotenoids, anthocyanins, brown pigments, and so on.

### Construction of the RPIOSL model for rice leaves

Most existing vegetation leaf radiative transfer models, such as PROSPECT and PIOSL models, simulate the radiative transfer of multiple vegetation leaves. Accurate simulation of leaf spectra can be achieved by entering the structural parameters and physical and chemical parameters of different species of vegetation leaves. In fact, the distribution of leaf structure and biochemical parameters of different types of vegetation is different [7, 15]. It is necessary to establish a radiation transfer model for a single vegetation leaf in order to understand the interaction mechanism between light and leaves. The traditional PROSPECT model assumes that the blade is homogeneous and the material inside is uniformly distributed. The PIOSL model proposed by Yu et al. [21] solves the problem of uneven distribution of absorbed substances inside the leaves. The blade interior is separated into two layers, and the material that absorbs light within the blade is organized into layers. When the electron microscope structure of rice leaves was scanned, it was found that there was a certain stratification phenomenon in rice leaves. Many scholars have also studied the morphology of rice leaves [17]. Rice leaves are composed of upper epidermis, mesophyll, and lower epidermis. In the cross section, epidermal cells are rectangular and epidermal and glandular hairs can be seen on the epidermis. Mesophyll is a green tissue composed of palisade tissue and sponge tissue. Palisade tissue cells are approximately rectangular columnar in shape, next to the upper epidermis, and vertically arranged with the upper epidermis. The cells are closely arranged, and there are more chloroplasts in the cells. Therefore, chlorophyll is more distributed in the upper layer of rice leaves. Sponge tissue is located under palisade tissue, which contains some irregular parenchyma cells, which are loosely arranged, with large gaps, and few cells in the chloroplast angle palisade tissue [34]. At different periods of rice growth and development, the internal structure of the rice leaves also changed slightly. It can be seen from the parameter optimization results for each rice period in Fig. 5 that the Cab and Cm in the rice leaves will change with the growth period transition. A deep-level capture of the distribution of material components in rice leaves will help to more accurately describe the radiation transmission process between light and rice leaves. Therefore, a more suitable radiative transfer model for rice leaves can be built by refining the leaf stratification and including additional elements in the stratification.

In this paper, by adjusting the parameters of the PIOSL model, the RPIOSL model which accords with the characteristics of rice leaf structure was constructed, which takes into account the internal structure of rice leaves. The model not only accurately simulates the reflectance spectrum of rice leaves but also conforms to the real internal structure of rice leaves, enhancing the interpretability of the model. In comparison to the PIOSL model, the biggest advantage of the RPIOSL model is that it is more suitable for radiation transfer simulation of rice leaves, optimizing the internal structure and elements of rice leaves by layers. The parameters of the RPIOSL model optimized by NSGA-II are consistent. We fixed the proportion of equivalent water thickness as 1. From the results of parameter optimization, we found that the proportion of Cab and Cm in rice leaves has a certain consistency. We will seek to construct a fixed proportion of Cab and Cm in rice leaves, which can shorten the training time of the model without affecting its accuracy. However, this paper only uses the data of one rice variety for verification, and in the future research, the differences of different rice varieties should be considered. Because there may be some differences in the internal structure of different rice varieties, such as the distribution of chlorophyll, water and dry matter.

## Conclusion

In this paper, the hypothesis of layered optical characteristics of rice leaf structure is put forward, and the RPIOSL model which can simulate the reflectivity spectrum of rice leaf is proposed by combining the PIOSL based on the hypothesis of layered optical characteristics of rice leaf with the structure of rice leaf.The model can simulate the reflectivity of rice leaves accurately by inputting the N of rice leaves, Cab, Cw and Cm. The structural parameters of RPIOSL model and the distribution ratio of chlorophyll and dry matter content in leaves were determined by NSGA-III algorithm.The structural parameters of the first layer of leaves were around 2, while those of the second layer were around 0–0.5. The ratio of Cab to Cw tended to be higher in the uppermost layer of the leaves. The four growth cycles of rice sample mulching are tillering stage jointing stage heading stage and grain filling stage, and the mean RMSE between the simulated spectra and the measured spectra of the RPIOSL and PROSPECT models were calculated in the full band and blue-green, yellow–red, and near-infrared bands, respectively. The findings indicate that the average RMSE of the constructed RPIOSL model is 0.1074, which is 0.0191 lower than the PROSPECT model. Among them, the spectral simulation effect of the RPIOSL model in the 560–780 nm band of yellow and red light is the best, and the RMSE at tillering period, jointing period, heading period and grouting period are 0.0584, 0.0576, 0.0724 and 0.0820, respectively, which are 0.0545, 0.0467, 0.0589 and 0.0603 lower than PROSPECT model, respectively. In future research, parameters such as nitrogen will be introduced to the RPIOSL model, providing more technical support for rice nitrogen inversion and top-dressing decision-making.

## Data Availability

The datasets used and analysed during the current study are available from the corresponding author on reasonable request.
